# Hydrophobic association supramolecular gel suitable for oil and gas drilling in fractured formation

**DOI:** 10.3389/fchem.2024.1468766

**Published:** 2024-11-26

**Authors:** Yongquan Han, Zhixue Yu, Zijun Guan, Rui Ma, Yuzhou Hu

**Affiliations:** ^1^ The Ninth Oil Extraction Plant of Changqing Oilfield Company, Xi’an, China; ^2^ Petrochina Liaohe Oil Production Technology Research Institute, Liaoning, China

**Keywords:** supramolecular gels, oil and gas drilling, viscoelastic properties, swelling properties, plugging performance

## Abstract

Supramolecular gel can be used to seal fractures and pores in the formation during oil and gas drilling and production. In this study, a supramolecular gel plugging agent based on hydrophobic association was prepared by free radical polymerization of acrylamide, octadecyl methacrylate, sodium dodecyl sulfate and other monomers by micellar copolymerization. The forming time, rheology, swelling, mechanical properties and plugging properties of supramolecular gels were studied. The experimental results show that the formation time of supramolecular gel plugging agent is controllable, and it has excellent viscoelastic recovery ability in a certain range of shear strain and scanning frequency. When the compression amount is close to 88.3%, the compressive stress reaches 3.82 MPa. Meanwhile, the swelling performance of supramolecular gel was good, and the equilibrium swelling degree reached 22.1% after 188 min swelling in 15% NaCl brine solution. In 1% NaCl solution, the equilibrium swelling degree of supramolecular gel reached 32.5%. In addition, supramolecular gel has strong formation bonding ability, good plugging performance, plugging rate is greater than 95%, can effectively reduce the leakage and improve the plugging effect of low permeability layer. It is of reference and guiding significance for the long-term plugging of heterogeneous and malignant leakage formations.

## 1 Introduction

Early plugging agents used in oil fields are polyacrylamides, acting by directing the flow of injected water after reservoir injection, diverting fluid flow into unaffected areas. They serve as relatively cost-effective treatment agents (Shaoqun et al., 2018; Lingmei et al., 2011). Breakthroughs have been continuously made in the research on plugging agents, with systematic studies on plugging agents suitable for complex reservoir formations, high temperatures, and high mineralization environments (Bing and Chao, 2021; Weiwei et al., 2021; Xiaoxiao et al., 2021; Shengqi et al., 2021; Fuyin et al., 2019). In recent years, commonly used chemical plugging agents in onshore oil and gas fields include polymer gel, particle, precipitation-type inorganic salts, and resin types. These agents exhibit effective sealing for high-permeability reservoirs in onshore oil and gas fields (Xinyu et al., 2022; Chunming et al., 2022; Fengbao et al., 2021; Xin et al., 2021; Rongchao et al., 2021; Wang, 2023; Tan et al., 2022; Zhenbin et al., 2017; Yang et al., 2022; Sun et al., 2020).

Gel-type plugging agents exhibit good plugging strength and controllable gelation time, making them suitable for various oil and gas fields with differing reservoir permeabilities, thus offering broad application prospects (Jinshneg et al., 2022; Yang et al., 2023; Yingrui et al., 2022). Ji et al. synthesized a water-soluble polymer-based plugging gel by incorporating special functional groups into the macromolecular chain (Yongzhong et al., 2015). This gel demonstrates unique efficacy in treating complex well leakages during high-pressure high-permeability gas well testing operations. To address the frequent leakage issue of oil-based drilling fluids in gas reservoirs within the Longmaxi formation in the Sichuan basin due to the formation of pores and microfractures, Yu et al. developed a high-temperature-resistant gel-type plugging agent for oil-based drilling fluids (Xin et al., 2021). This agent exhibited good plugging performance at well W204H. Cheng et al. successfully developed a polyacrylamide/citric acid/chromium gel system with low initial viscosity, pH responsiveness, and controllable gelation strength and cross-linking time (Jiecheng et al., 2020). Through deep profile control field testing at the Daqing oil field, it was verified that this gel system effectively plugs advantageous flow channels in deeper parts of the reservoir, demonstrating promising results for high permeability formations. To overcome the shortcomings of conventional chemical gel plugging agents in terms of poor high-temperature resistance and mechanical properties, Bai et al. synthesized a hybrid cross-linked gel plugging material (Yingrui et al., 2021), which satisfies the pressurized plugging requirements for high-permeability fractured leaky formations. Wang suggests that the mechanical strength of pure polymer gels is relatively low (Jianjun et al., 2005), whereas the mechanical strength of new multifunctional composite gels is notably high, effectively addressing leakage issues in deep well formations. Wang developed a new supramolecular plugging material (Yong et al., 2018) with the capability to adapt to different formation depths. It forms gels with different strengths and elasticities under different temperatures, exhibiting wide adaptability.

In recent years, supramolecular gels have attracted wide attention due to their excellent adaptability, adjustability and mechanical properties, and have potential application prospects in many fields (Chang et al., 2016). Jiang et al. developed a supramolecular gel system with certain strength that can form in the leakage layer within a controllable time (Jiang et al., 2016), which plugs pores with a size of 0.15–1.5 mm and a pressure of more than 7.5 MPa. Wang et al. developed a supramolecular gel plugging agent (GP-A) (Wang et al., 2021), which is different from the linear gel of HPAM in that it is mainly spiderweb star-shaped connection, and the addition of chromium ions can enhance the bonding effect of the microstructures of the gel interconnects, showing high mechanical strength. Sun et al., 2022; Jiang et al., 2022. systematically introduced the application status of supramolecular gels in oil and gas drilling and production engineering fields, such as drilling plugging and enhancing oil recovery, combined with the applicability and action mechanism of supramolecular gels (Jinshneg et al., 2022), and proposed the development direction of supramolecular gels in oil and gas drilling and production engineering in the future. Tuncaboylu et al. copolymerized the macromolecular hydrophobic monomers octadecyl methacrylate and dodecyl acrylate with the hydrophilic monomer acrylamide in the micelle of sodium dodecyl sulfate (Tuncaboylu et al., 2011), and the supramolecular gel prepared through strong hydrophobic association has good self-healing properties and high toughness (Hongbin et al., 2023).

Supramolecular gels possess a unique dynamic reversible network structure, making them adaptable to complex oil and gas reservoir environments. They exhibit excellent mechanical properties and hold promising application prospects in the oil and gas drilling and production industry. In this study, a supramolecular gel based on hydrophobic association was prepared by micellar copolymerization with acrylamide (AM) as the main chain monomer and 2-acrylamide-2-methylpropanesulfonic acid (AMPS) as the high temperature resistant monomer. It demonstrates excellent viscoelastic properties and adaptive plugging performance, rendering it suitable for long-term plugging of severe loss circulation reservoirs in the oil and gas drilling process.

## 2 Experimental section

### 2.1 Experimental materials

Reagents: Acrylamide (AM, 99%) and 2-acrylamido-2-methylpropane sulfonic acid (AMPS, 98%), both of analytical grade, were purchased from Aladdin Chemical Reagent Co., Ltd. Methyl methacrylate (SMA, 96%) and sodium dodecyl sulfate (SDS, 99%), also of analytical grade, were obtained from Shanghai Macklin Biochemical Co., Ltd. Sodium sulfate anhydrous (SSA, 99%), sodium chloride (NaCl, 99.8%), and potassium persulfate (KPS, 99.9%), all of analytical grade, were sourced from Sinopharm Chemical Reagent Co., Ltd.

Equipment: The experiments were conducted using a range of equipment, including a TGA/DTA thermogravimetric analyzer (METTLER TOLEDO Company), HAAKE MARS 60 model high-temperature high-pressure rotational rheometer (Thermo Fisher company, Germany), TIR-7600 Fourier-transform infrared spectrometer (Shanghai Precision Instrumentation Co., Ltd.), DF-101T collective thermal magnetic stirrer (Shanghai Neue Instrument Co., Ltd.), Digital constant temperature water bath (Shaoxing Jingmai Instrument and Equipment Co., Ltd.), CMT4000 universal testing machine (Jinan New Test Gold Testing Machine Co., Ltd.), Quanta200F field emission scanning electron microscope (Thermo Fisher, Germany), and a high-temperature and high-pressure plugging and leakage replacement device (Nantong Xinhua Cheng Scientific Research Instrument Co., Ltd.).

### 2.2 Preparation of supramolecular gel

Firstly, acrylamide and 2-acrylamide-2-methylpropanesulfonic acid were dissolved in deionized water, then sodium dodecyl sulfate was added, and fully stirred into a homogeneous solution at 300 r/min. Secondly, octadecyl methacrylate was added, mixed at 500 r/min, then potassium persulfate was added, continued to stir for 2 h, and quickly degassed in an inert atmosphere (nitrogen), and transferred to the reaction vessel after degassing; Finally, supramolecular gel can be prepared by placing the reaction vessel in an oven at 60°C for 6 h.

### 2.3 Infrared analysis

The chemical structure of the supramolecular gel was tested by Fourier transform infrared spectrometer (Nicolet IS50 FTIR). Before testing, the supramolecular gel was cleaned with deionized water to remove the unreacted part of the supramolecular gel, and then the supramolecular gel was ground to powder after drying in a vacuum oven. The samples were prepared by potassium bromide pressing method. The infrared spectrum scanning range was 4000–550 cm^−1^, the scanning temperature was 25°C–250°C, the resolution was 1 cm^−1^, and the scanning times was 8.

### 2.4 Thermogravimetric analysis

The thermal stability of chemical bonds in supramolecular gel powders was detected by thermal analyzer (TGA550, United States). The supramolecular gel was dehydrated in an oven at 105°C. During each measurement, 10–15 mg of supramolecular gel sample was placed in a sealed pan and the sample was heated from 40°C to 600°C at a rate of 10°C/min. The experiment was carried out in a nitrogen atmosphere of 50 mL/min.

### 2.5 Field emission scanning electron microscope

The microstructure of supramolecular gels was characterized by Hitachi S-4700 field emission scanning electron microscope (FESEM). The lyophilized supramolecular gel was obtained by freezing the gel in liquid nitrogen and then freeze-dried at −50°C and 30 × 10^-3^ mbar pressure for 48 h. Lyophilized supramolecular gels were injected into liquid nitrogen and carefully fractured to obtain new cross sections, which were then mounted on aluminum roots and sprayed with a thin layer of gold for scanning, and SEM imaging was performed at 10kv.

### 2.6 Rheological properties

The rheological properties of supramolecular gel samples were tested by HAAKE MARS 60 high temperature and high pressure rotary rheometer. The type of flat plate rotor used in the experiment is P35/Ti (rotor diameter is 35 mm). The temperature of the test sample should be balanced for at least 30 min, and the temperature error should be controlled within ±0.1°C. The thickness and diameter of supramolecular gel samples were 1 mm and 35 mm, respectively. In this study, at constant frequency (1 Hz), supramolecular gel samples were subjected to strain scanning tests at different temperatures (20°C and 60°C) and different shear stresses (τ = 10 Pa, 30 Pa, 50 Pa, 80 Pa, 100 Pa), and the strain range was γ = 0.1%–1,000%. The sample of supramolecular gel was tested by frequency scanning under fixed strain (γ = 1%). The frequency variation range was 0–20 Hz. The energy storage modulus (G′) and loss modulus (G″) of supramolecular gel were determined by frequency scanning.

### 2.7 Compression test

The supramolecular gel was made into a cylinder with a bottom diameter of 20 mm and a height of 10 mm. The electronic universal testing machine (CMT4000 electronic universal testing machine, Shenzhen Xinsui Material Testing Company) was used to test the compressive mechanical properties of the supramolecular gel at room temperature. The compression speed was set as 5 mm/min, and the stress-strain curves of the gel samples during compression were recorded. To ensure the accuracy of the data, each test group was repeated three times.

### 2.8 Swelling test

Swelling kinetics were performed by soaking the weighed and dried hydrogel samples in their respective saline solution. The dynamics of supramolecular gel volume as a function of time was studied until the equilibrium swelling ratio was reached. Measurements were made under different conditions with reference to saline with different ionic strengths, pH and temperatures. The expansion rate of supramolecular gel is calculated by Formula 1 and Formula 2, where W_t_ is the mass of supramolecular gel after swelling for a certain time, W_d_ is the mass of supramolecular gel after drying, and We is the mass of supramolecular gel after swelling and equilibrium.

$$Q_{t}=\left(W_{t}-W_{d}\right)/W_{d}$$ (1)

$$Q_{eq}=\left(W_{e}-W_{d}\right)/W_{d}$$ (2)

### 2.9 Plugging performance test

The plugging performance of the supramolecular gel was evaluated using a high-temperature and high-pressure plugging and displacement device. An artificial core, with a length of 7 cm and a diameter of 2.5 cm, was selected to measure its porosity and permeability. It was then placed into the core holder, and the prepared supramolecular gel system was injected into the holder. The change in injection pressure was observed, and when the injection volume reached 3.0 PV and the injection pressure did not significantly change, the injection was stopped, and the pressure was recorded. After the supramolecular gel system was formed, gas was injected into the holder, and the pressure change at the injection port was recorded. When the injection pressure reached its highest breakthrough point and gradually stabilized, the breakthrough pressure was recorded, indicating the plugging strength of the supramolecular gel.

## 3 Preparation and formulation optimization of supramolecular gel

### 3.1 Supramolecular gel formulation optimization

**FIGURE 1:**
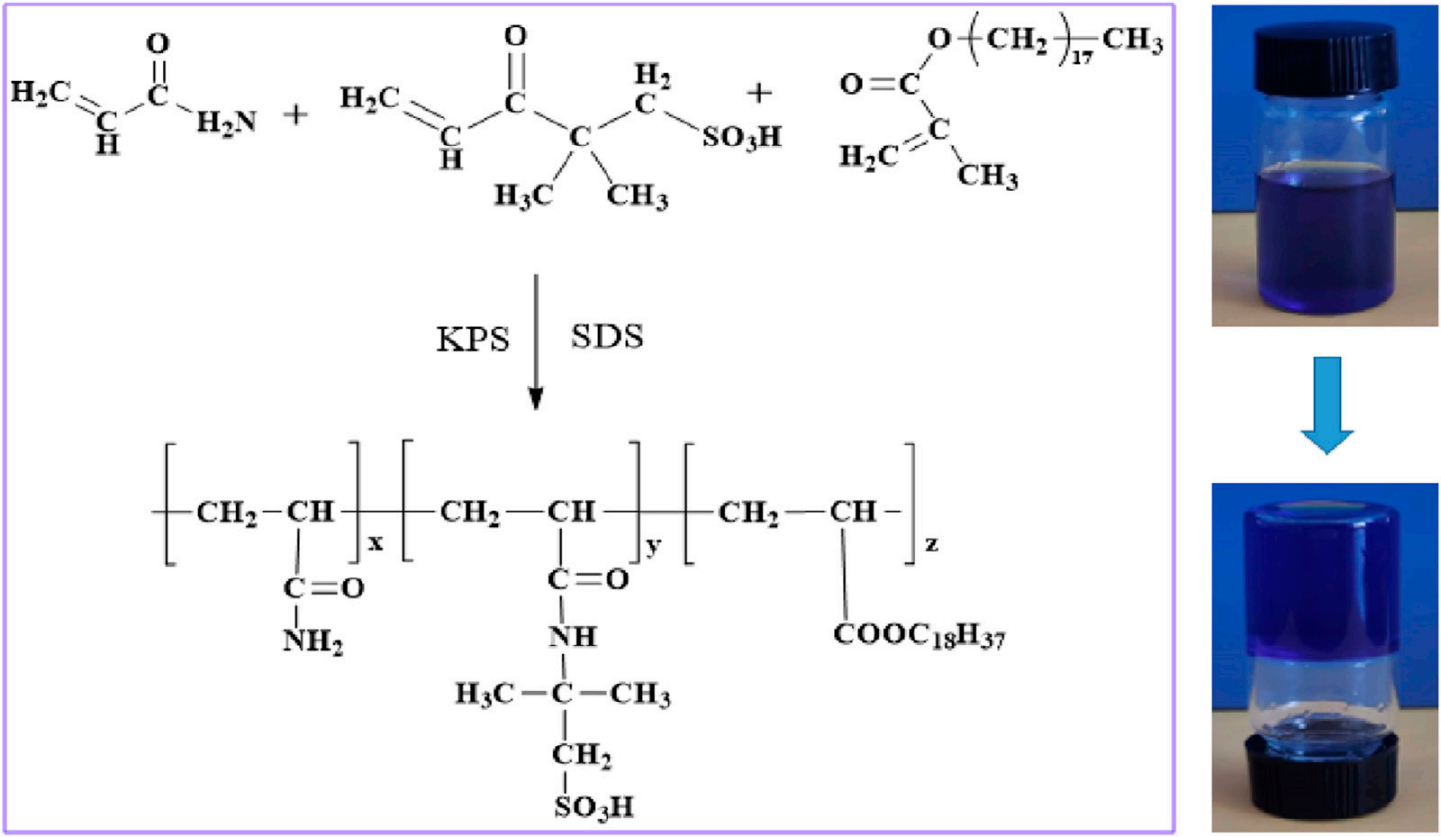
Supramolecular gel molecular structure formula.

As an example, the preparation of 200 g of supramolecular gel solution involves several steps. First, 85 g of deionized water is poured in five separate beakers. Subsequently, five portions of 24 g of acrylamide (24 g, 0.3377 mol), 4 g of 2-acrylamido-2-methylpropanesulfonic acid (4 g, 0.0214 mol), 0.8 g of octadecyl methacrylate (0.8 g, 0.0021 mol), and 0.2 g of potassium persulfate (0.2 g, 0.0007 mol) are individually weighed. Additionally, 1.0 g, 2.0 g, 3.0 g, 4.0 g, and 5.0 g of sodium dodecyl sulfate are weighed accordingly. The acrylamide and 2-acrylamido-2-methylpropanesulfonic acid are dissolved into deionized water first, followed by the addition of sodium dodecyl sulfate. The mixture is stirred thoroughly at 300 r/min until a homogeneous solution is formed. Subsequently, octadecyl methacrylate is added and stirred well at 500 r/min. Finally, potassium persulfate is added, and stirring is continued for 2 h. The solution is then quickly degassed in an inert atmosphere (nitrogen) and transferred to the reaction vessel after degassing. The reaction vessel is placed in an oven at 60°C for a constant temperature reaction. The molecular structure formula of supramolecular gel is shown in Figure 1.

**FIGURE 2:**
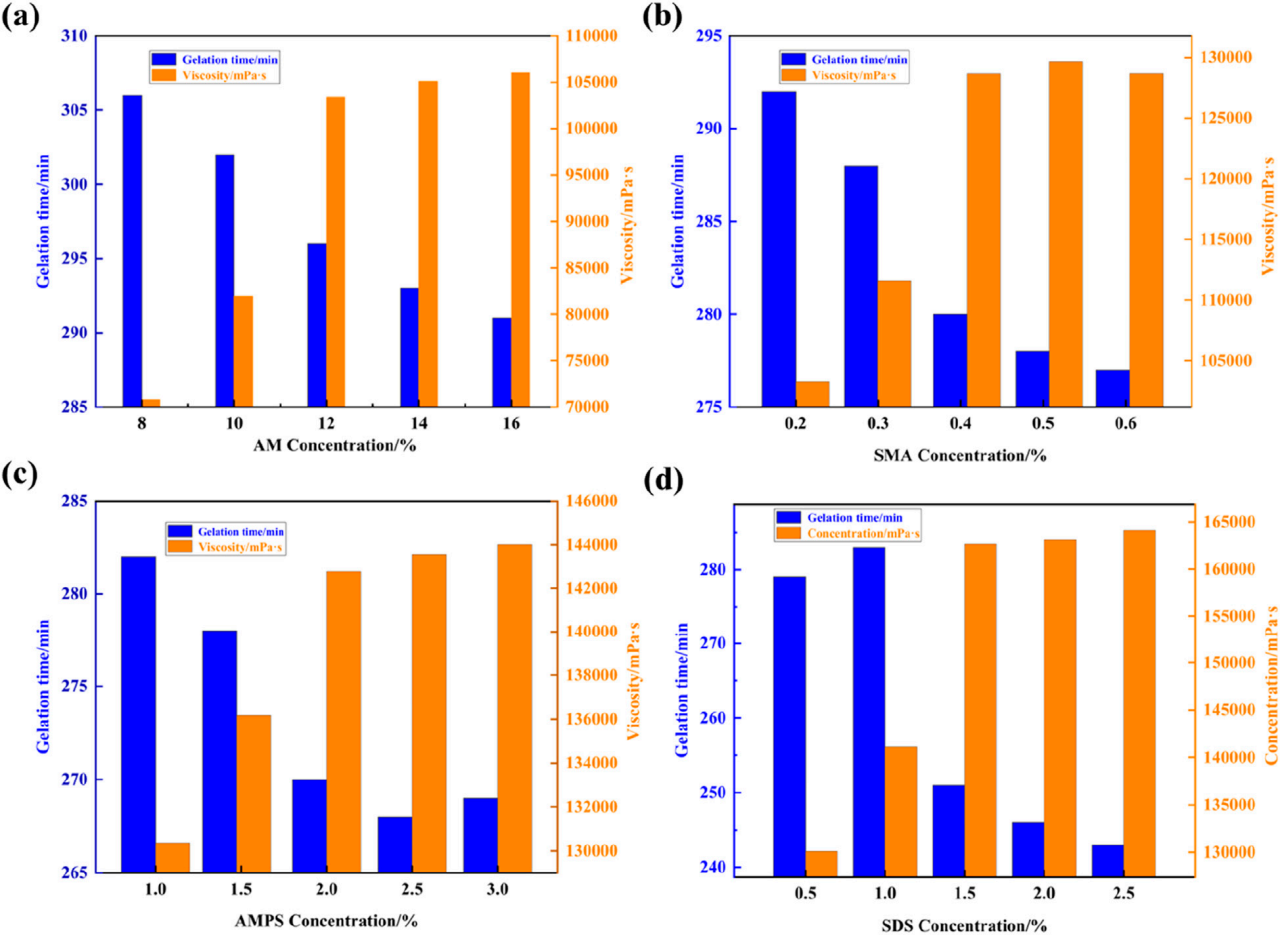
Effects of the concentration of supramolecular gel synthetic components on gelling time and viscosity: **(A)** AM, **(B)** SMA, **(C)** AMPS, and **(D)** SDS.

The optimal formulation ratio for synthesizing supramolecular gels was determined using the univariate analysis method, as depicted in Figure 2. Figure 2A illustrates that the gelation time gradually decreases with increasing concentration of AM. Upon reaching a concentration of 12% AM, both the gelation time and viscosity are optimized, showing no significant variations with further concentration increases. Thus, the optimal AM concentration is determined to be 12%. Similarly, varying the concentration of SMA revealed that the gelation viscosity of supramolecular gels reaches optimal values at a concentration of 0.4% SMA, with further increases not significantly affecting viscosity (Figure 2B). Figure 2C demonstrates that at a concentration of 2% AMPS, there are no significant variations in gel viscosity, indicating optimal gelation time. Finally, Figure 2D displays the experimental results of varying the concentration of the surfactant SDS, showing that gel viscosity increases with surfactant usage and eventually stabilizes. The most optimal viscosity of the supramolecular plugging agent is achieved at a 1.5% SDS concentration. This result can be attributed to the method of preparation using micellar copolymerization, where surfactants form aggregates in water, solubilizing hydrophobic monomers to form micelles. This process enables copolymerization of hydrophobic and water-soluble monomers, consequently increasing viscosity. However, as surfactant usage increases, hydrophilic and hydrophobic monomers undergo sufficient copolymerization to form a dense polymer gel network structure, which can no longer be solubilized into micelles by the surfactant. As a result, viscosity reaches an optimal point and may slightly decrease thereafter. Therefore, the optimal formula for preparing supramolecular gel plugging agents consists of 12% AM, 2% AMPS, 0.4% SMA, and 1.5% SDS.

### 3.2 Effects of initiator on the gelation condition of supramolecular gel plugging agents

The polymerization reaction of supramolecular gels is aqueous solution free-radical polymerization, free-radical polymerization reactions releases heat, initiators undergo decomposition reaction under external heating conditions, releasing free-radicals. The decomposition reaction of the initiator is shown in Formula 3 and Formula 4:

I→2R, (3)

$$R_{d}=-\frac{d\left[I\right]}{dt}=k_{d}\left[I\right],$$ (4)

where I is the initiator molecule; *R* is the primary radical; *R*_*d*_ is the initiator decomposition rate, mol·(L·s)^−1^; K_d_ is the rate constant of the initiator, s^-1^; and [I] is the initiator concentration, mol·L^−1^.

**FIGURE 3:**
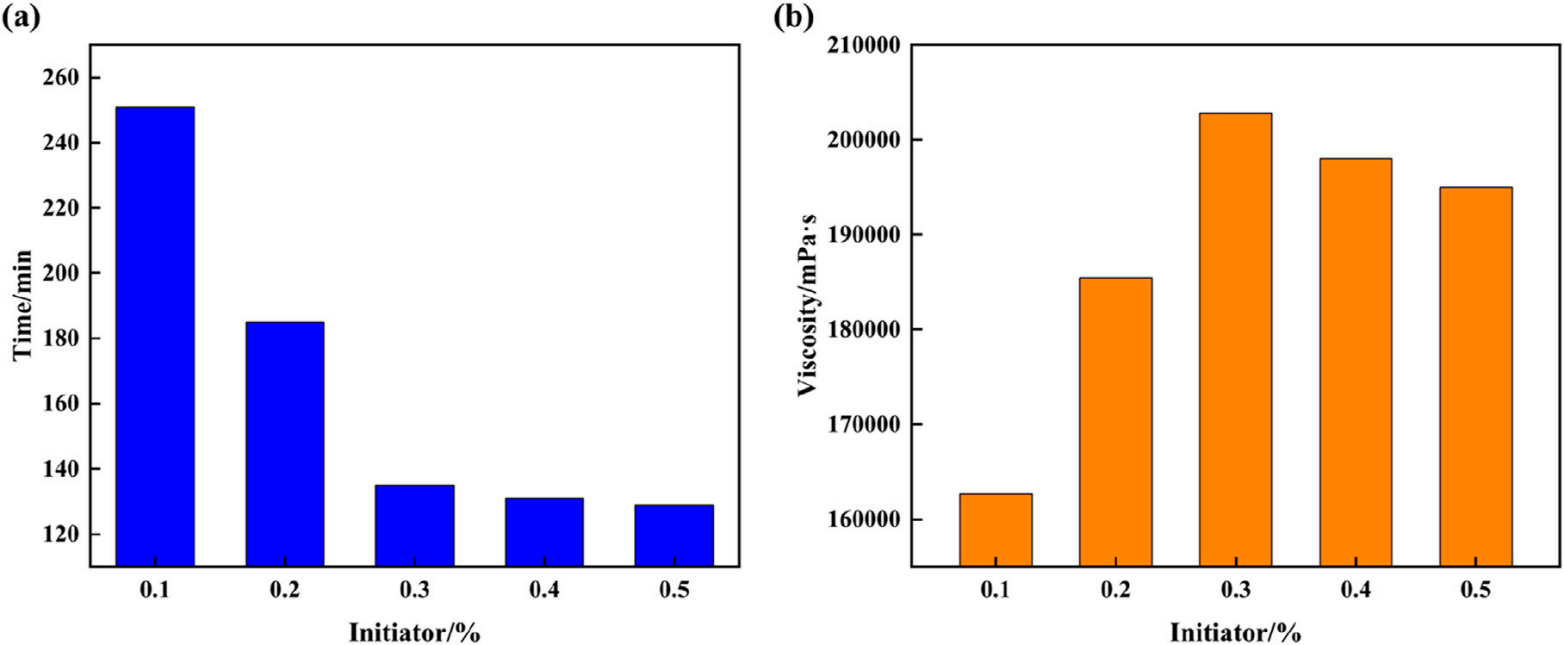
Plot of the influence of initiator on gelation time and viscosity of supramolecular gel plugging agent: **(A)** Gelation time; **(B)** Gel viscosity.

The laboratory initiator system used to synthesize supramolecular gels is potassium sulfate/sodium bisulfite; this system not only undergoes redox reactions during polymerization but also reduces the activation energy needed by the free-radicals, which enables polymerization reaction to continue even under low-temperature environments. However, the decomposition rate of the initiator system in high-temperature formations is too high, where decomposition occurs without initiating the formation of supramolecular gels. To understand the mechanisms of the influence of initiators on the gelation condition of supramolecular gels, different initiator dosages are designed to study the variation conditions of gelation time and viscosity. It can be seen from Figure 3 that the time required for the supramolecular gel to go from a flowable liquid state to a tough solid state is different for different amounts of added initiator. With an increase in the initiator dosage, the gelation time of supramolecular gels decreases, and the viscosity increases first and then decreases. When the initiator dosage is 0.3%, the gelation time is 135 min; at this point, the viscosity of the supramolecular gel can reach a maximum of 202,781 mPa·s. This is primarily because initiator molecules decompose under a certain temperature condition, which breaks covalent bonds and produces free radicals in the process. The formation of free radicals increases the cross-linked reaction efficiency, speeding up the polymerization reaction in the system and promoting the transition of the gel from liquid to solid state. Additionally, within a specific range, the greater the amount of active groups, the faster the system polymerization reaction.

### 3.3 Effect of temperature on the gelation condition of supramolecular gel plugging agents

**FIGURE 4:**
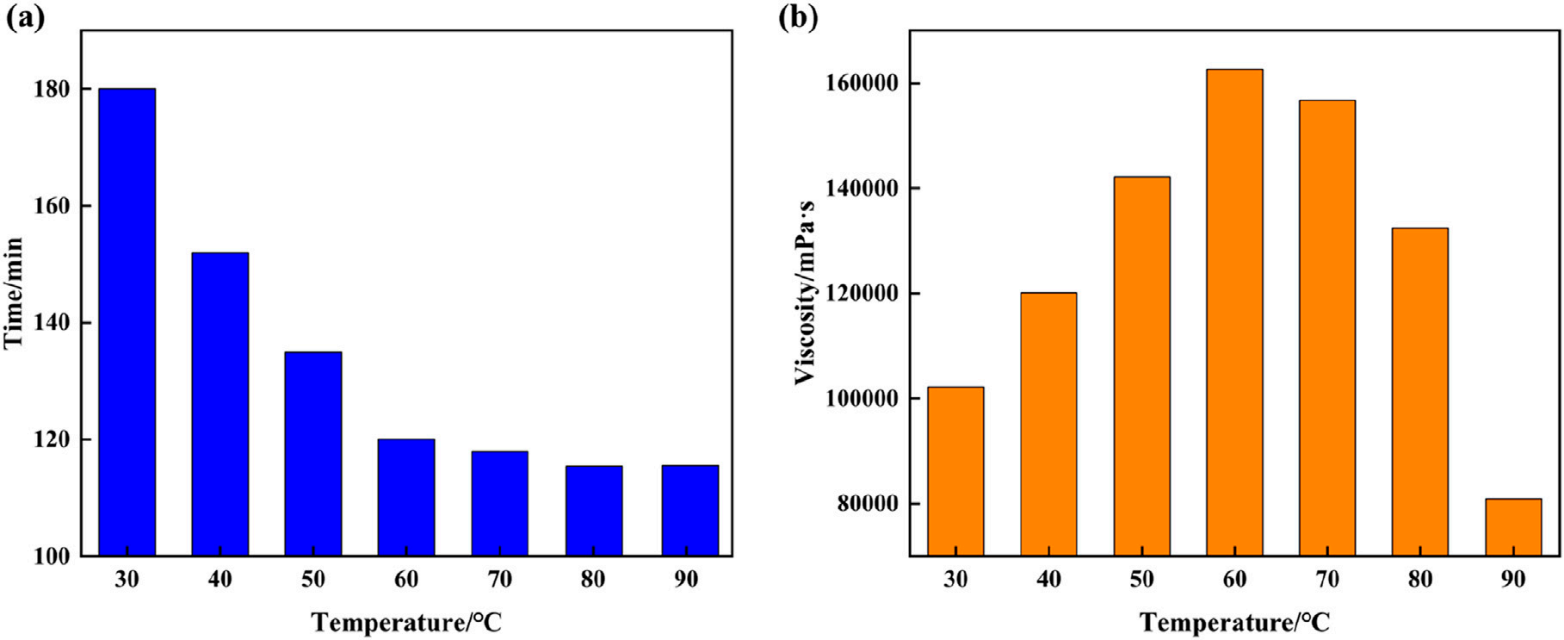
Plot of the effect of temperature on the gelation time and viscosity of supramolecular gel plugging agents: **(A)** Gelation time, **(B)** Gel viscosity.

The optimal solution for synthesizing supramolecular gels is 12% AM + 2% AMPS + 0.4% SMA + 1.5% SDS + 0.3% initiator. To study the effects of temperature on the gelation time and viscosity of supramolecular gel plugging agents, the gelation time and viscosity of supramolecular gels synthesized using this formulation are observed at 30°C–90°C. As the experimental results indicate in Figure 4, the gelation time of supramolecular gels gradually decrease with the increase in temperature, with a significant reduction in gelation time when temperatures are lower than, and the magnitude of change above 60°C is less. It can be seen from changes in viscosity under different temperatures that the viscosity of supramolecular gels initially increases with temperature and then decreases, with the gel viscosity reaching the maximum value of 162,663 mPa·s at 60°C. The reason for the above phenomenon is that increases in temperature accelerate free radical polymerization of hydrophilic and hydrophobic monomers of supramolecular gels, which triggers the surfactant to quickly solubilize the hydrophobic monomers and form micelles, which increases polymerization viscosity. However, when the temperature is too high, the spatial network structure of the supramolecular gel will be destroyed, which can break the non-covalent bonds formed by self-assembly, resulting in a corresponding decrease in the gel viscosity. Therefore, the optimal reaction temperature of supramolecular gels is 60°C, and the gelation time of the supramolecular gel under this temperature condition is 120 min.

In summary, it can be seen that the supramolecular gel has been successfully prepared with an optimal synthesis temperature of 60°C, the optimal preparation formula is AM12% + AMPS2% + SMA 0.4% + SDS1.5% + 0.3% initiator, the gelation time is 120 min, and the gel viscosity is 202,781 mPa·s. Supramolecular gels can be used as plugging material, and can be applied to seal pores and cracks in formations in oil and gas drilling engineering. Therefore, evaluation of the physiochemical properties of supramolecular gels is helpful for the research on its mechanisms and for the popularization of its application.

## 4 Performance evaluation of supramolecular gel plugging agents

### 4.1 Structural characterization of supramolecular gel plugging agents

**FIGURE 5:**
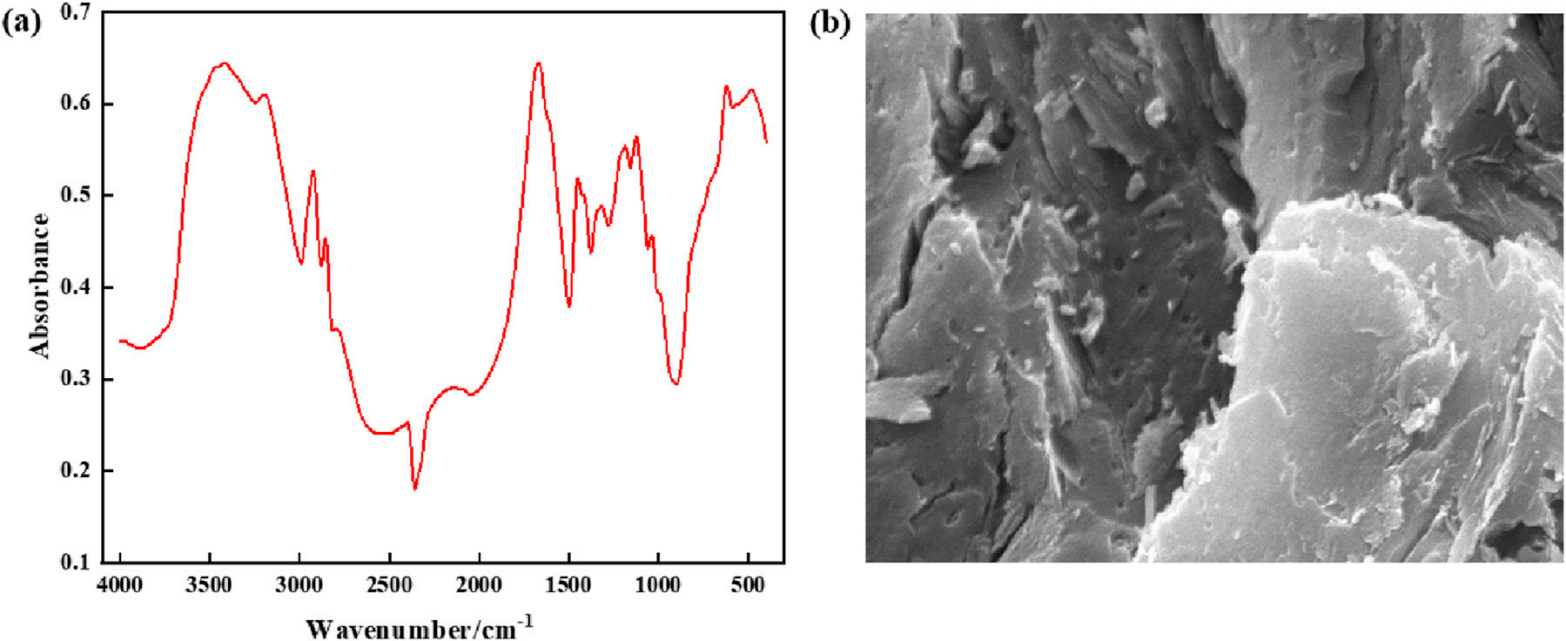
Structural characterization of supramolecular gels: **(A)** Infrared spectra; **(B)** Microstructure.

The supramolecular gel plugging agent prepared in this study is a high-strength gel material based on hydrophobic binding and hydrogen bonding, with excellent properties. To demonstrate the successful preparation of supramolecular gels, the chemical functional groups of the gel materials were characterized by Fourier transform infrared spectroscopy (FTIR-7600). Results from infrared spectroscopy in Figure 5 show that the signal intensity close to the characteristic peaks of 3,412 cm^−1^ and 3,197 cm^−1^ is high, indicating that this region includes N-H stretching vibration in polyacrylamide, O-H stretching vibration in the hydroxyl group, and O-H stretching vibration in adsorbent water. Additionally, due to the occurrence of multi-molecular bonding, intermolecular hydrogen bonds are formed and peaks are superimposed with a wider peak shape. Meanwhile, the characteristic peak of the carbon-oxygen double bond (C=O) stretching vibration at 1,676 cm^−1^ has a high intensity and sharp peak, which indicates the presence of amide and carboxylic acid groups; further, intermolecular hydrogen bonding easily occurs due to multi-molecular binding. There are large and wide peaks between 2,700 and 2000 cm^−1^, indicating that the prepared supramolecular gel materials contain hydroxyl (-OH) chemical groups that can form hydrogen bonds. Micro-structural characterization of the supramolecular gel is performed using the Quanta200F field emission scanning electron microscope (FESEM), and the magnification is 10,000 times, indicating that supramolecular gels have a densely layered network structure with strong binding between layers. Therefore, the infrared spectroscopy performed above confirms that supramolecular gels have been successfully synthesized, with components tightly bound together with relatively strong hydrophobic binding and hydrogen bonding interactions.

### 4.2 Rheological properties of supramolecular gel plugging agents

**FIGURE 6:**
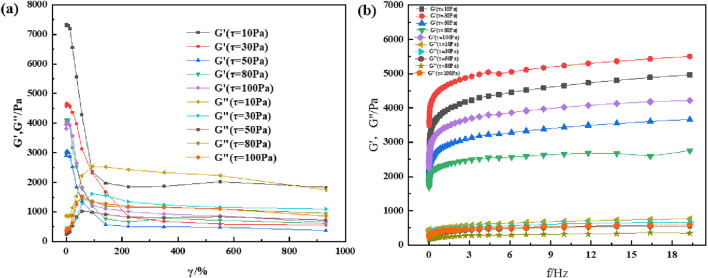
Rheological properties of supramolecular gels under room-temperature (20°C): **(A)** Variation of modulus with shear strain under constant shear stress; **(B)** Variation of modulus with shear frequency under constant shear stress.

**FIGURE 7:**
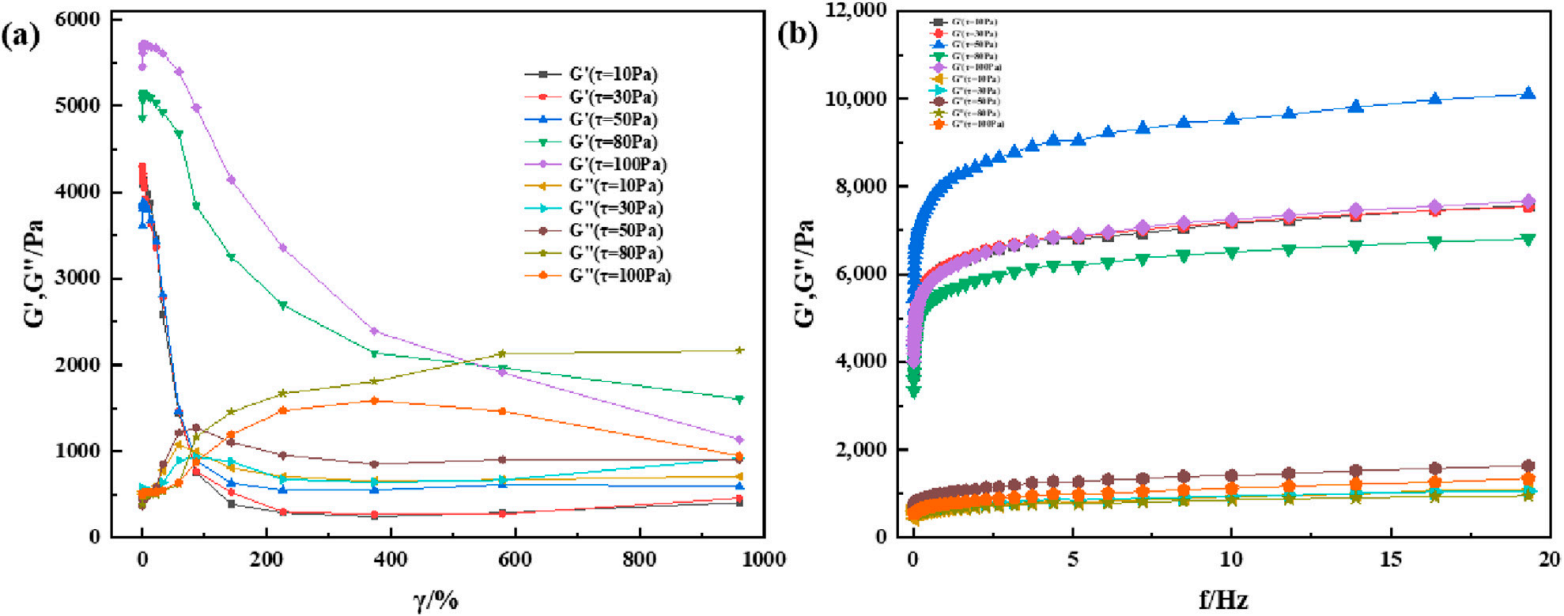
Heological properties of supramolecular gels under room-temperature (60°C): **(A)** Variation of modulus with shear strain under constant shear stress; **(B)** Variation of modulus with shear frequency under constant shear stress.

Most viscoelastic materials are subjected to varying degrees of stress for a given application. There is always a shear limit for any given application, and optimal viscoelastic properties are maintained according to the desired results. Therefore, the material must remain homogeneous and maintain its structure at high shear loads. Rheology is a technique for characterizing the properties of viscoelastic materials, and variations in the modulus with changes in shear strain and frequency is commonly used for characterizing and comparing viscoelastic properties of materials. To understand the rheological properties of supramolecular gels, the HAAKE MARS 60 high-temperature high-pressure rotational rheometer is used to perform shear strain and frequency scanning tests on supramolecular gel specimens; the experimental results are shown in Figures 6, 7. The results show that the rheological properties of supramolecular gels are affected by the change of shear strain and frequency. It can be seen from Figure 6A that supramolecular gel begins to undergo sol-gel phase transition when the strain value is greater than 80%, which is represented by a decrease in the energy storage modulus and an increase in the loss modulus. Finally, its energy storage modulus remains near 1000 Pa. As can be seen from Figure 6B, supramolecular gel has a certain dependence on frequency. In the frequency scanning range, the energy storage modulus and loss modulus both show an upward trend with the increase of scanning frequency, and the energy storage modulus is always greater than the loss modulus, showing good rheology. It can be seen from Figure 7A that the sol-gel phase transition of supramolecular gels has a similar change rule when the temperature is 60°C, both of which are manifested as a decrease in the energy storage modulus and an increase in the loss modulus. At the same time, it can be seen from Figure 7B that with the increase of scanning frequency, the energy storage modulus and loss modulus both show an upward trend. However, it can be seen by comparing Figures 6, 7 that temperature has a certain effect on the viscoelasticity of the material; the higher the temperature, the greater the storage modulus and loss modulus. Further, the corresponding elastic properties weaken. This was also observed by experts and scholars in prior studies, who believe that with the increase in temperature and frequency, it is difficult for the macromolecular chains present in the gel specimen to rearrange into a regular network structure, which results in the dehydration and hardening of the gel under higher temperature and shear loads, causing the gel to loosen the solid-liquid phase transition ability and weakening the corresponding elastic properties (Jianjun et al., 2005). This is similar to the conclusion drawn from the supramolecular gels in this study, and further illustrates the excellent viscoelastic recovery ability of supramolecular gels within a certain range of shear strain and scanning frequency.

### 4.3 Swelling properties of supramolecular gel plugging agents

Gel swelling is a multifaceted process comprising three sequential stages: first, water molecules penetrate the interior of the gel; second, relaxation of polymer chains within the gel; third, the polymer network expands throughout the water, inducing the gel to swell. To examine the swelling characteristics of supramolecular gels, these gels are first desiccated in an oven to a constant weight and then weighed. Thereafter, the dried gels are immersed in NaCl solutions of varying concentrations, and the mass of the gels at swelling equilibrium is recorded. The extent of swelling of supramolecular gels under diverse temperatures and salt concentrations is quantified using Formula 5 and Formula 6:

$$S_{R}=\frac{\left(M_{t}-M_{d}\right)}{M_{d}},$$ (5)

$$S_{eq}=\frac{\left(M_{e}-M_{d}\right)}{M_{d}},$$ (6)

where $M_{t}$ denotes the mass of the supramolecular gel after a specified period of swelling, $M_{d}$ denotes the mass of the dried supramolecular gel, $M_{e}$ represents the mass of the supramolecular gel after swelling equilibrium.

**FIGURE 8:**
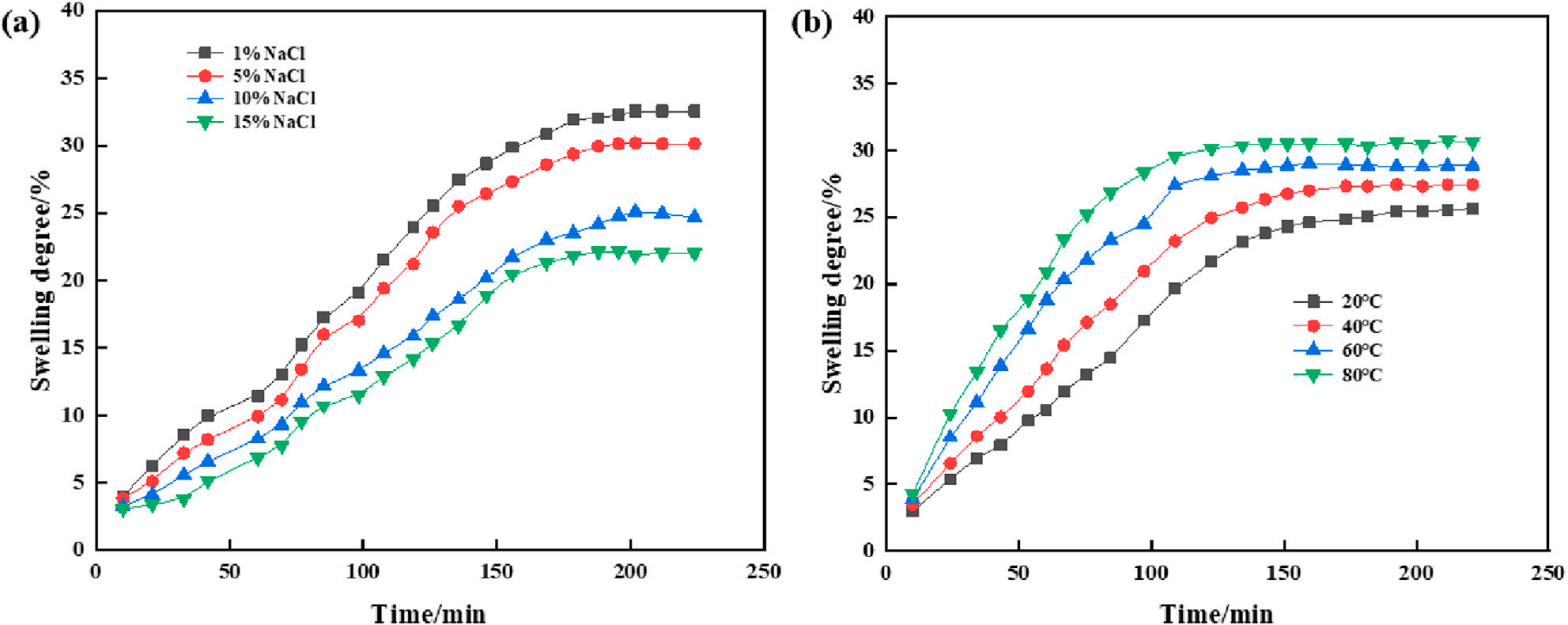
Swelling properties of supramolecular gels: **(A)** Effects of NaCl solution concentration on swelling properties; **(B)** Effects of temperature on swelling properties.

Figure 8 llustrates the impact of varying salt concentrations and temperatures on the swelling behavior of supramolecular gels. As observed, the salt concentration significantly influences the swelling properties; the swelling degree of the gel in 1% NaCl solution is greater than that in 15% NaCl saline solution. Specifically, the equilibrium swelling percentage of the gel in 15% NaCl solution after 188 min is 22.1%, whereas in 1% NaCl solution, it reached 32.5%. This variation suggests that the diffusion of sodium and chloride ions rapidly increases the concentration gradient, thereby accelerating the swelling process. The swelling of supramolecular gel is the result of the equilibrium between the diffusion rate of water in the gel network and the interaction between the polymer network. When the gel is placed in saline water, ions in the solution (such as NaCl) enter the gel network, reducing the osmotic pressure difference between the inside and outside of the gel, i.e., reducing the driving force of expansion. As the salt concentration increases, the osmotic pressure decreases further, making it more difficult for water molecules to enter the gel, thus reducing the swelling degree of the gel.

Additionally, temperature also affects the swelling dynamics of supramolecular gels. In Figure 8B, a 10% saline solution was used to study the effect of temperature on the swelling properties of supramolecular gels. The data indicate a progressive increase in swelling extent with rising temperature, attributable to the enhanced diffusion of the polymer network within water and improved polymer–solvent interactions, which consequently accelerate the swelling of supramolecular gels.

The swelling kinetics of supramolecular gel was studied in order to further clarify the effect of temperature and salt concentration on swelling behavior. The swelling of dry hydrogel in water is described by the swelling kinetics Formula 7, which is as follows:

$$S_{R}/S_{eq}=K_{s}t^{n}$$ (7)

In the formula, Ks is the characteristic constant of the gel, n is an important parameter describing the swelling mechanism of the material, it determines the diffusion type, and reflects the relationship between the diffusion rate of the solvent and the relaxation rate of the polymer chain. Generally speaking, there are three kinds of solvent diffusion in the gel: when n ≤ 0.5, the diffusion of the solvent meets Fickian diffusion, which is called Fickian diffusion (type I); When n = 0.5–1.0, the solvent diffusion rate is equivalent to the relaxation rate of macromolecular chains, which is called non-fickian diffusion (type II). When n ≥ 1.0, it is the relaxation diffusion process of macromolecular chains, which is called relaxation diffusion (type III).

Taking the logarithm of both sides of Formula 8 yields:

$$\mathrm{lg}\left(\frac{S_{R}}{S_{eq}}\right)=\mathrm{lg}K_{s}+n\text{lgt}$$ (8)

**FIGURE 9:**
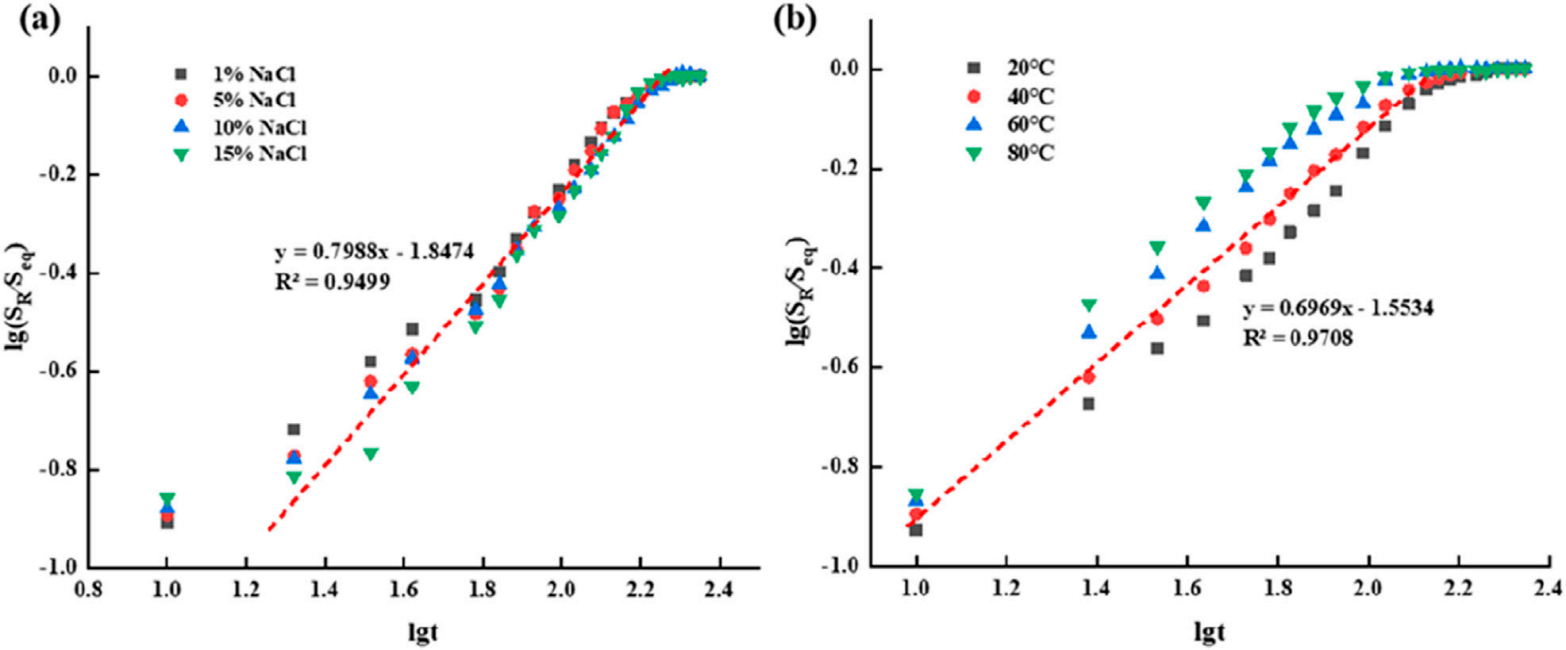
Fitting curve of swelling kinetics of supramolecular gel **(A)** Fitting curve of swelling in salt solutions with different concentrations of NaCl; **(B)** Swelling fitting curves at different temperatures.

That is, $\mathrm{lg}\left(S_{R}/S_{eq}\right)$ is linear to lgt , where n is the slope and lgK_s is the intercept. Linear fitting was carried out according to the experimental data in Figure 8, and the fitting curve obtained was shown in Figure 9. Fick characteristic index n and fitting related factors of supramolecular gel can be further calculated by fitting curves, as shown in Table 1.

**TABLE 1:** Swelling kinetic constants of supramolecular gels.

Influencing factor	Fick characteristic index (n)	Intercept	Fit the correlation factors (R2)
NaCl salt concentration	0.7988	−1.8474	0.9499
Temperature	0.6969	−1.5534	0.9708

According to the fitting results, we found that the Fick characteristic index n of salt solutions with different concentrations and under different temperature conditions is between 0.5 and 1.0, indicating that the swelling process of supramolecular gel is non-fickian diffusion type (type II), so the diffusion rate of small molecules can be ensured to be the same as the relaxation rate of large molecules before and after the phase transition temperature. Besides the diffusion of water molecules, the gel swelling process is greatly influenced by the relaxation of gel network and polymer segment, the interaction between water molecules and polymer network, and the interaction between polymer network groups.

### 4.4 Mechanical properties of supramolecular gel plugging agents

**FIGURE 10:**
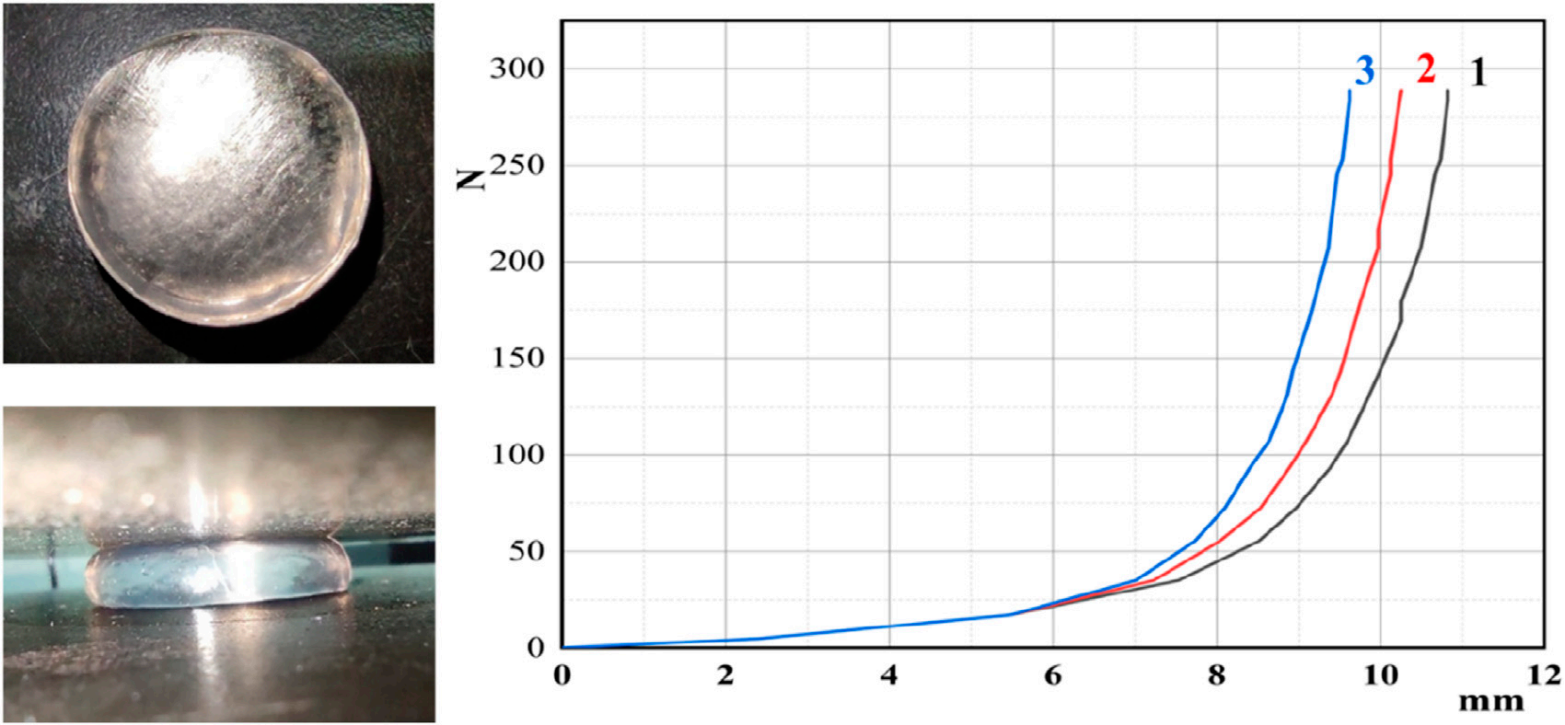
Compressive properties of supramolecular gels.

To investigate the compressive properties of supramolecular gels, the gel was shaped into a cylinder with a base diameter of 10 mm and a height of 12 mm. The CMT4000 universal testing machine measured the compressive mechanical properties at 25°Cwith a compression speed set at 5 mm/min. The same supramolecular gel sample was compressed three times in the experiment. Compressibility is the ratio of the compressed amount to the height in the original state. As depicted in Figure 10, the supramolecular gel can be compressed by approximately 10.6 mm, achieving nearly 88.3% compression. At this level of compression, the stress reached 3.82 MPa, and no visible cracks were observed on the surface of the gel, indicating robust compressive properties of the gel.

This phenomenon is attributed to the cross-linking points formed by hydrophobic monomers; the long, straight alkyl chains exhibit low steric hindrance. Their structure is similar to the surfactant SDS used in the synthesis of the gel, enabling effective solubilization into the SDS micelles. Consequently, a high concentration of hydrophobic monomers enhances the linkage strength, allowing a denser and more resilient gel network structure with excellent compression resistance.

### 4.5 Plugging performance of supramolecular gel plugging agents

The plugging performance of supramolecular gels was assessed using a high-temperature, high-pressure plugging and displacement device. An artificial rock core (length: 7 cm; diameter: 2.5 cm) was selected. The porosity and permeability of the core were determined prior to the experiment. The core was secured in the core gripper, and the prepared supramolecular gel system was injected into this core. During the injection, which continued until the injection volume reached 3.0 pore volumes (PV) without significant pressure changes, the pressures were meticulously recorded. After gelation, gas was introduced into the core gripper and the injection port pressure changes were monitored until the pressure stabilized at the maximum breakthrough point, indicating the plugging strength of the supramolecular gel.

According to the experimental design principle, the initial breakthrough pressure and pressure gradient before the effective formation of the supramolecular gel sealing layer are zero. When the supramolecular gel is gelled and then injected into the gas, the corresponding breakthrough pressure and pressure gradient value will be generated. The difference between the core samples used in the experiment is mainly due to the difference in porosity and permeability. Porosity is the ratio of the sum of all pore space volumes in a rock sample to the volume of the rock sample, which reflects the size of the pore space in the rock. The greater the porosity, the greater the pore space in the rock, which may allow more fluid to pass through. Permeability is an important index to measure the difficulty of fluid flow in rock pore space, and porosity is one of the key factors affecting permeability. During the plugging operation, the plugging agent needs to effectively fill the pore space in the rock to form an effective plugging barrier. The pore space inside the core with larger porosity is more complex and extensive, which puts forward higher requirements for the distribution of plugging agent. If the plugging agent does not fully fill all the pore Spaces, or a weak plugging layer is formed in some areas, the plugging effect will be greatly reduced. As listed in Table 2, the permeability of all three tested cores significantly decreased after plugging with the supramolecular gel. The plugging rates exceeded 95%, thereby demonstrating the effective plugging capabilities of the gel. Notably, for high-permeability reservoir formations, both the breakthrough pressure and pressure gradient increased after plugging, suggesting superior plugging effects in these scenarios, which is crucial for preventing drilling fluid leakage in gas reservoirs under high formation pressures.

**TABLE 2:** Plugging effect of supramolecular gel system.

Core sample	Length/cm	Diameter/cm	Porosity/%	Permeability without plugging/μm^2^	Permeability with plugging/μm^2^	Plugging rate/%	Breakthrough pressure/kPa	Breakthrough pressure gradient/MPa/m
XQ-1	7	2.5	19.32	0.1925	0.0096	95.01	241.6	3.45
XQ-2	7	2.5	22.84	0.4306	0.0146	96.61	525.3	7.51
XQ-3	7	2.5	26.36	0.9124	0.0231	97.47	824.6	11.78

Supramolecular gels are increasingly recognized for their exceptional adaptability, tunability, and mechanical properties, offering promising application prospects across various fields. Unlike traditional covalently bonded polymer gels, supramolecular gels are composed of networks formed through self-assembly mediated by non-covalent interactions, including hydrogen bonding, hydrophobic interactions, host–guest interactions, and electrostatic forces. These interactions facilitate reversible structural configurations and contribute to the high-performance characteristics of the gel. Minor variations in these non-covalent interactions can shift the equilibrium conditions of the gel system, prompting solid–liquid phase transitions that significantly improve the mechanical properties of the supramolecular gels.

**FIGURE 11:**
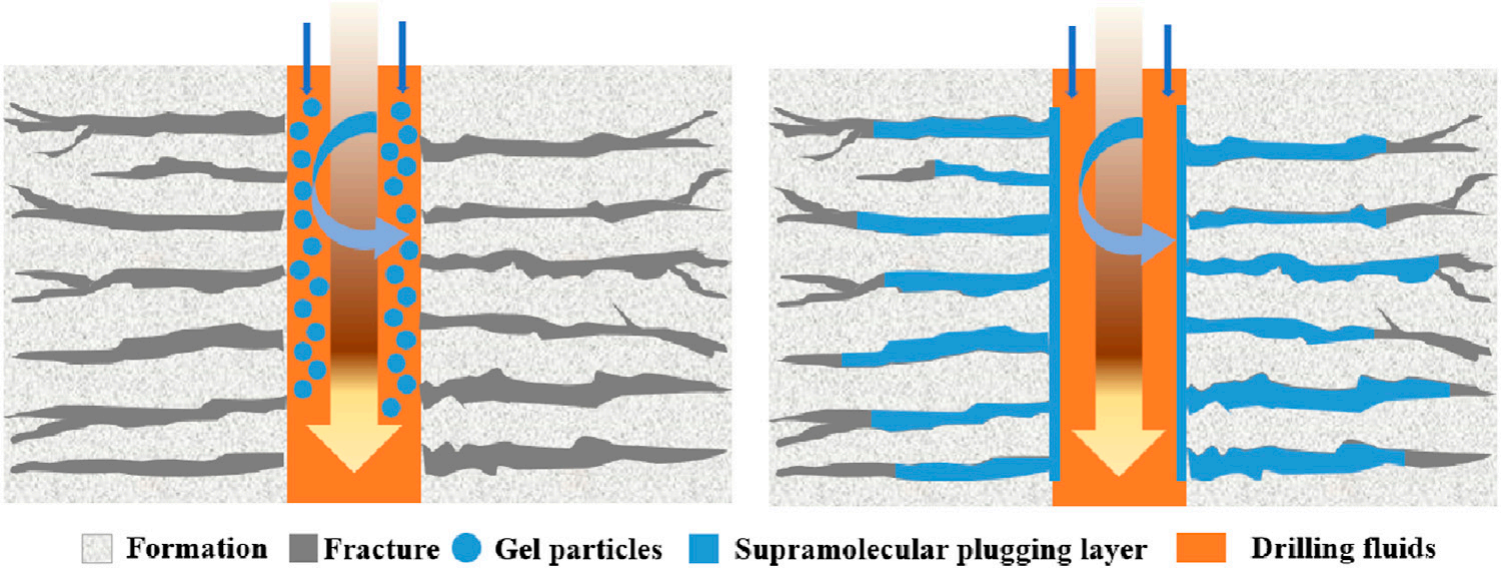
Mechanism of supramolecular gel plugging.

Supramolecular gels synthesized via hydrophobic binding can be effectively utilized as plugging materials. The molecular structure of these gels is specifically designed to exhibit excellent heat-resistant and viscous properties, thereby overcoming the limitations associated with conventional polymer flooding. After entering the formation, the gel adheres to the surface of porous rock media, which increases flow resistance and obstructs the dominant water flow channels (Figure 11). The interaction of hydrogen bonds and electrostatic forces between the surface ion groups of the gel imparts high-strength and self-healing capabilities that facilitate the formation of durable sealing layers and act as effective gel barriers.

## 5 Conclusion

(1) The ideal composition for supramolecular gel plugging agents includes 12% acrylamide, 2% 2-acrylamido-2-methylpropanesulfonic acid, 0.4% octadecyl methacrylate, 1.5% sodium dodecyl sulfate, and 0.3% potassium persulfate. The optimal plugging performance of these gels was observed when prepared at 60°C with a gelation time of 120 min and a gel viscosity of 202,781 mPa∙s.
(2) The developed supramolecular gel plugging agent displays commendable rheological and mechanical properties. It possesses an excellent viscoelastic recovery ability within a defined range of shear strains and scanning frequencies. The compressive stress of the gel reaches 3.82 MPa at approximately 88.3% compression, with no visible cracks on the surface of the supramolecular gel.
(3) The supramolecular gel demonstrates effective swelling characteristics, achieving an equilibrium swelling of 22.1% after 188 min in a 15% NaCl saline solution, and 32.5% in a 1% NaCl saline solution. These properties indicate its capability to expand and fill geological formations efficiently.
(4) The gelation time of the supramolecular gel plugging agents is adjustable, which enables their application across fracture lose channels of various sizes. They exhibit robust stratigraphic bonding and excellent plugging performance, with plugging rates exceeding 95%. This renders them highly suitable for application in diverse and complex leakage scenarios such as cracks, cavities, and pores.

## Data Availability

The original contributions presented in the study are included in the article/supplementary material, further inquiries can be directed to the corresponding author.
